# A Study on Associations of Long Noncoding RNA HOTAIR Polymorphisms With Genetic Susceptibility to Chronic Kidney Disease

**DOI:** 10.1002/jcla.25086

**Published:** 2024-07-03

**Authors:** Mahdi Majidpour, Ramin Saravani, Saman Sargazi, Sara Sargazi, Mahdiyeh Harati‐Sadegh, Shadi Khorrami, Mohammad Sarhadi, Ali Alidadi

**Affiliations:** ^1^ Clinical Immunology Research Center Zahedan University of Medical Sciences Zahedan Iran; ^2^ Cellular and Molecular Research Center Research Institute of Cellular and Molecular Sciences in Infectious Diseases, Zahedan University of Medical Sciences Zahedan Iran; ^3^ Department of Clinical Biochemistry, School of Medicine Zahedan University of Medical Sciences Zahedan Iran; ^4^ Genetics of Non‐Communicable Disease Research Center Zahedan University of Medical Sciences Zahedan Iran; ^5^ Metabolic Syndrome Research Center Mashhad University of Medical Sciences Mashhad Iran; ^6^ Department of Nephrology, Faculty of Medicine Zahedan University of Medical Sciences Zahedan Iran

**Keywords:** chronic kidney disease, gene polymorphism, HOTAIR, long noncoding RNA

## Abstract

**Background:**

The importance of long noncoding RNAs (lncRNAs) in various biological processes has been increasingly recognized in recent years. This study investigated how gene polymorphism in HOX transcript antisense RNA (HOTAIR) lncRNA affects the predisposition to chronic kidney disease (CKD).

**Methods:**

This study comprised 150 patients with CKD and 150 healthy controls. A PCR‐RFLP and ARMS‐PCR techniques were used for genotyping the five target polymorphisms.

**Results:**

According to our findings, rs4759314 confers strong protection against CKD in allelic, dominant, and codominant heterozygote genetic patterns. Furthermore, rs3816153 decreased CKD risk by 78% when TT versus GG, 55% when GG+GT versus TT, and 74% when GT versus TT+GG. In contrast, the CC+CT genotype [odds ratio (OR) = 1.66, 95% confidence intervals (CIs) = 1.05–2.63] and the T allele (OR = 1.50, 95% CI = 1.06–2.11) of rs12826786, as well as the TT genotype (OR = 2.52, 95% CI = 1.06–5.98) of rs3816153 markedly increased the risk of CKD in the Iranian population. Although no linkage disequilibrium was found between the studied variants, the C_rs12826786_T_rs920778_G_rs1899663_G_rs4759314_G_rs3816153_ haplotype was associated with a decreased risk of CKD by 86% (OR = 0.14, 95% CI = 0.03–0.66).

**Conclusion:**

The rs920778 was not correlated with CKD risk, whereas the *HOTAIR* rs4759314, rs12826786, rs1899663, and rs3816153 polymorphisms affected the risk of CKD in our population. It seems essential to conduct repeated studies across various ethnic groups to explore the link between *HOTAIR* variants and their impact on the disease outcome.

## Graphical abstract

A study of the association between HOTAIR polymorphisms and the risk of chronic kidney disease (CKD) was carried out on 300 subjects using both ARMS‐PCR and PCR‐RFLP methods. Visualization of gel electrophoresis patterns and data analysis showed a positive association between rs12826786 and rs1899663 and CKD risk and a negative association between rs4759314 and susceptibility to this chronic disease.

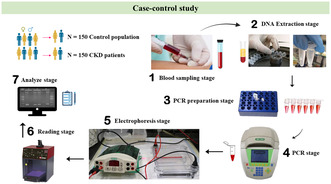

## 1 Introduction

Chronic kidney disease (CKD) represents a significant public health challenge worldwide, with a multifaceted etiology influenced by genetic, environmental, and lifestyle factors [1, 2]. The increasing prevalence of CKD affirms the need for a deeper understanding of its molecular underpinnings to facilitate early detection and targeted therapeutic interventions [3]. Among the various contributors to CKD risk, emerging evidence points toward the role of genetic polymorphisms, particularly within long noncoding RNA (lncRNA) genes [4, 5].

In recent years, lncRNAs have emerged as key players in various physiological and pathological processes [6]. Aberrant regulation of cell functioning by lncRNAs has become one of the factors of various diseases [7, 8]. LncRNAs are involved in the regulation of various processes at a genetic and epigenetic level. They can be used as lures, conduits, supports, and signaling switches in the cellular signaling processes [9]. Differences in structural parameters of lncRNAs—including length, secondary structures, and number of exons—facilitate their performance of various functions in both the nucleus and cytoplasm. Multiple regulatory axes involving various miRNAs have been identified in diseased cells for extensively studied lncRNAs like HOX transcript antisense intergenic RNA (HOTAIR), Metastasis Associated Lung Adenocarcinoma Transcript 1 (MALAT1), and taurine‐upregulated gene 1 (TUG1). The lncRNA/miRNA axis has been found to play a crucial role in suppressing immune responses in patients, making it a promising target in addition to established molecular pathways [10]. Some unique lncRNAs specific to the renal cell types and their upregulation contributed to the pathological changes involved in FSGS development, such as LOC105375913 and LOC105374325. The microarray study found that the upregulation of LOC105375913 in tubular cells of patients diagnosed with focal‐segmental glomerulosclerosis (FSGS) was mediated by the C3a/p38/XBP‐1s pathway to cause profibrosis through sequestering miR‐27b and overexpression of Snail. Likewise, the up‐regulation of LOC105374325 expression in the apical podocytes of these patients is also well correlated with programmed cell death of podocytes through sequestration of miR‐34c and miR‐196a/b, which are negative regulators of pro‐apoptotic factors in kidney [11].

Among lncRNAs, HOTAIR has garnered attention for its involvement in conditions like renal cancer [12], Type 2 diabetes mellitus (T2DM) [13], coronary artery disease [14], and hypertension [15]. Accumulating evidence has indicated that lncRNA HOTAIR plays a crucial role in approximating chromatin‐modifying complexes to target sites on the genome; HOTAIR has been identified to be expressed in various kidney cell types and upregulated in Diabetic Kidney Disease (DKD) and high glucose‐exposed podocytes. The results of the bioinformatic analysis indicated that the HOTAIR expression level was positively correlated with genic neighbor homeobox C11 (HOXC11), which is involved in the developmental patterning but whose contribution to the adult kidney remains questionable [16]. A comparison of the transcriptional level between CKD biopsy samples and control kidney RNA samples indicated that HOTAIR was overexpressed in CKD cases. This result confirms the hypothesis that HOTAIR is associated with the development of CKD and may act as an indicator for its diagnosis or as a therapeutic target [16, 17]. Furthermore, it has been demonstrated that suppression of *HOTAIR* can increase miR‐124 to inhibit the Notch1 pathway and thus reduce epithelial‐to‐mesenchymal transition (EMT) of kidney cells; hence, HOTAIR can be a therapeutic target for renal interstitial fibrosis, which is considered a pathological characteristic of CKD [18].

The human *HOTAIR* gene is located within the *HOXC* locus on chromosome 12q13.13. The HOTAIR RNA transcript, which is 2.2 kilobases in length, undergoes splicing, polyadenylation, and 5′‐capping. It extends across approximately six exons and operates as a trans‐acting lncRNA. The *HOX* gene clusters, comprising genes from *HOXA* through *HOXD*, encode transcription factors that regulate gene expression in cis, with HOTAIR identified initially as the first trans‐acting lncRNA in this context. HOTAIR does not affect the transcription of HOXC cluster genes despite co‐expression. Instead, HOTAIR acts in Trans, recruiting repressive protein complexes to the *HOXD* cluster on chromosome 2, leading to the epigenetic silencing of genes within this cluster. Specifically, HOTAIR's role in the developmental stages of mammals involves silencing key genes, including tumor suppressors and metastasis suppressors, within the *HOXD* cluster. Notably, Cell proliferation can be inhibited by downregulating cyclins and CDKs, which are essential for cell cycle progression, by *HOXD* genes, including *HOXD3*, *HOXD8‐10*, and *HOXD12* [19]. In addition to well‐established genes and proteins implicated in the modulation of apoptosis, including p53, P21, and caspases, substantive evidence demonstrates the pivotal role of HOTAIR in cell death [20, 21].

Variations in *HOTAIR* expression, both downregulation and upregulation, have been observed in diverse cancer types [22], and single nucleotide polymorphisms (SNPs) located in the *HOTAIR* encoding gene have been recently associated with several multifactorial diseases [7, 8, 23]. Given the impact of gene polymorphisms on gene expression, it becomes crucial to investigate the association between gene polymorphisms and the occurrence of diseases.

SNPs in lncRNAs are implicated in many diseases including kidney diseases. Discovering potential genetic changes in lncRNA HOTAIR related to CKD may have given valuable information on how the pathogenesis of kidney disease is initiated and may have opened doors to new diagnostic and therapeutic methods.

**SCHEME 1:**
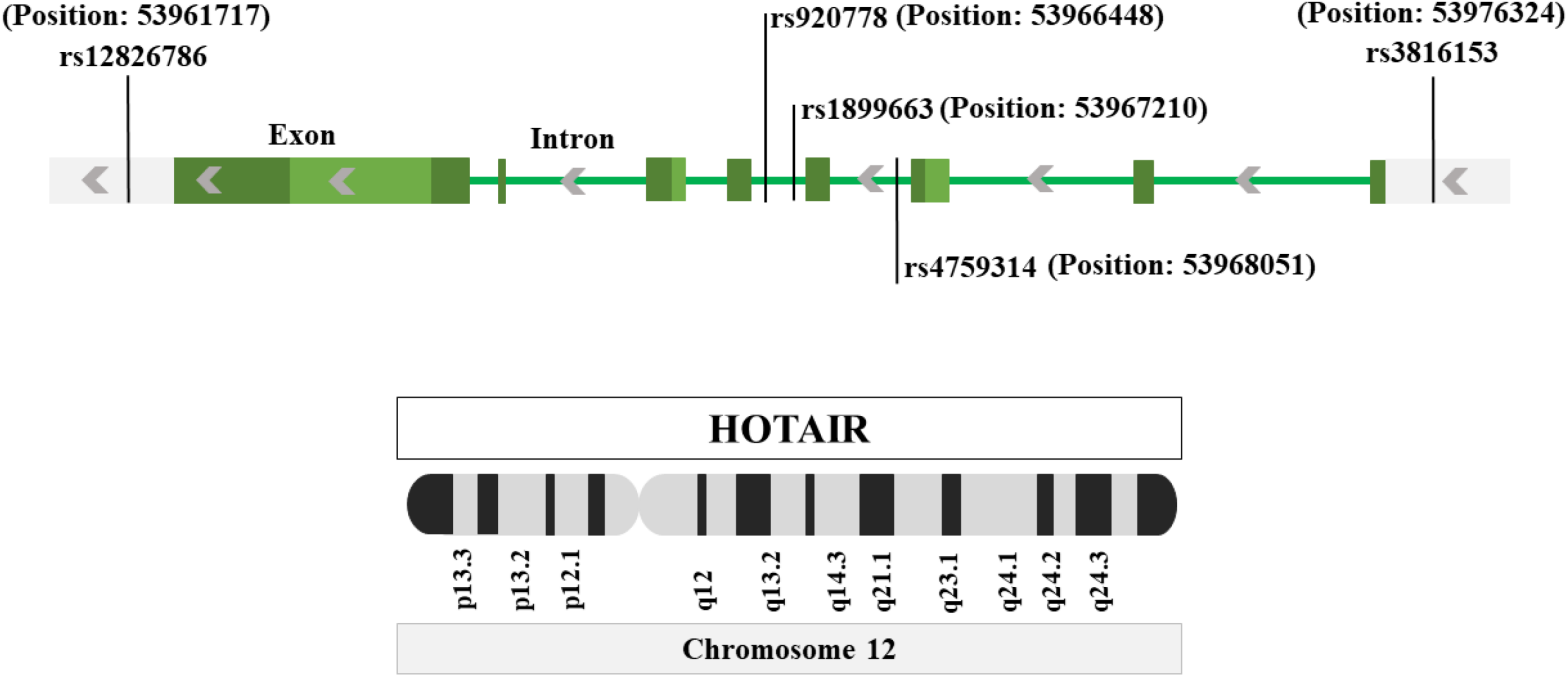
Location of *HOTAIR* polymorphisms located on chromosome 12.

Due to the lack of research evidence on the mechanism underlying the relation between genetic variants of *HOTAIR* and CKD risk, in this study, we aimed to investigate the role of specific polymorphisms within the *HOTAIR* gene—namely, rs4759314, rs920778, rs12826786, rs1899663, and rs3816153 (Scheme 1)—in the pathogenesis of CKD. Exploring such genetic factors becomes increasingly crucial as they may serve as potential biomarkers for early CKD detection and contribute to personalized treatment strategies.

## 2 Materials and Methods

### 2.1 Study Design

This case–control study comprised 300 Iranian participants, including 150 CKD cases admitted to the Bu‐Ali Hospital, Zahedan, Iran, between February and December 2022, as well as 150 healthy controls admitted to the same clinic for routine checkups. Inclusion criteria for the patient group followed Kidney Disease Improving Global Outcomes (KDIGO) guidelines, defined by a reduction in Glomerular Filtration Rate (GFR) and with or without a record of proteinuria [24]. CKD stages were classified into five levels of estimated glomerular filtration rate (eGFR), as mentioned earlier [1]. Exclusion criteria included pregnant women, patients who had undergone kidney transplantation previously, invasive tumors, polycystic kidney disorder, acute inflammation, acute kidney disease, and any other complications or systemic diseases. The case controls were randomly chosen from individuals without proteinuria or kidney or renal disorders (GFRCKD‐EPI >90 mL/min per 1.73 m^2^) [25]. For biochemical examinations, 2 mL of the whole blood test was drawn through phlebotomy and collected in serum gel tubes. One milliliter of venous blood was also collected in tubes containing ethylenediaminetetraacetic acid (EDTA) for genomic DNA isolation.

### 2.2 Laboratory and Clinical Analysis

We recorded body mass index (BMI), age, gender, and biochemical parameters for the studied groups. Table 1 compares CKD patients and healthy controls on demographic and clinical features. Biochemical assay kits were all provided by Pars Azmoun‐Co (Iran) and were used to measure blood urea nitrogen (BUN), total cholesterol (TC), serum creatinine, and fast blood glucose (FBS) according to the manufacturer's protocol.

**TABLE 1:** Clinical and demographic attributes of the study population.

Parameter evaluated	CKD (*n* = 150) (mean ± SD)	Controls (*n* = 150) (mean ± SD)	*p*‐value*
Gender			0.512
Female	59	71	
Male	91	79	
Age (year)	52.13 ± 16.74	49.64 ± 14.81	0.293^a^
BMI (kg/m^2^)	27.09 ± 4.08	24.44 ± 4.38	**<0.001** ^**a**^
BUN (mg/dL)	34.18 ± 18.91	12.52 ± 5.16	**<0.001** ^**a**^
Serum creatinine (mg/dL)	2.14 ± 1.37	1.39 ± 0.99	**<0.001** ^**a**^
Albuminuria (%)			
<30 mg/g	75 (50.0)	—	—
30–300 mg/g	56 (37.3)	—	—
>300 mg/g	19 (12.7)	—	—
Stage (based on GFR)			
Stage I	45 (30.0)	—	—
Stage II	33 (22.0)	—	—
Stage III	49 (32.7)	—	—
Stage IV	15 (10.0)	—	—
Stage V	8 (5.3)	—	—

Abbreviations: BMI, body mass index; BUN, blood urea nitrogen; CKD, chronic renal insufficiency‌.

^a^ Mann–Whitney test.

* *p* < 0.05 is statistically significant between CKD patients and healthy controls (bold *p*‐values).

### 2.3 DNA Extraction, Genotyping, and SNP Selection

DNA extraction from circulating blood was performed utilizing the salting‐out strategy [24]. The *HOTAIR* gene polymorphisms (rs4759314, rs920778, rs12826786, rs1899663, and rs3816153) were genotyped using both allele‐specific amplification refractory mutation system‐polymerase chain reaction (ARMS‐PCR) and polymerase chain reaction‐restriction fragment length polymorphism (PCR‐RFLP) methods. PCR‐RFLP is considered one of the most precise and selective methods of identifying genetic variations in different organisms. It is very selective, can detect very low‐frequency SNPs, yields results faster than many other genetic analysis methods, is not too technically demanding, and is very reliable. On the other hand, the ARMS‐PCR technique is very effective in achieving a faster and more efficient amplification of the required allele containing the target SNP. Besides, this approach is commonly used because it does not require expensive restriction enzymes, unlike in the PCR‐RFLP technique. We utilized the National Center for Biotechnology Information (NCBI) database to identify these five SNPs with a minor allele frequency (MAF) greater than 0.1. To amplify the SNPs, Primer sequences shown in Table 2 were designed using Gene Runner 3.05 software and then synthesized using Gen Fan Avaran Company (Iran).

**TABLE 2:** The primers used for genotyping of *HOTAIR* gene polymorphisms.

SNP	Genotyping method	Primer sequence	Annealing temperature (°C)	Restriction enzyme	Product size (bp)
rs920778 C/T	PCR‐RFLP	F: 5′‐TTACAGCTTAAATGTCTGAATGTTCC‐3′ R: 5′‐GCCTCTGGATCTGAGAAAGAAA‐3′	56	*MspI*	T: 140 C: 113 + 27
rs4759314 A/G	PCR‐RFLP	F: TTCAGGTTTTATTAACTTGCATCAGC R: ACCCAAAACCATTTCCTGAGAG	55	*AluI*	G allele: 124 A allele: 98 + 25
rs12826786 C/T	ARMS‐PCR	F: AGACCTTGGTCCAATTCC R (G allele): AGAGGGAAGGAGCTTAGGATAAACG R (A‐allele): AGAGGGAAGGAGCTTAGGATAAACA	62	—	364
rs1899663 G/T	ARMS‐PCR	F (C‐allele): AAAGCCTCTAATTGTTGTCATC F (A‐allele): AAAGCCTCTAATTGTTGTCATA R: AGACCCTCAGGTCCCTAATA	54	—	127
rs3816153 G/T	PCR‐RFLP	F: CTCCAGGCAGGCTAGCACCG R: CTCCAGGCAGGCTAGCACCG	63	*AluI*	G: 276 T: 255 + 21

Abbreviations: ARMS‐PCR, amplification refractory mutation system polymerase chain reaction; F, forward; R, reverse; RFLP‐PCR, restriction fragment length polymorphism polymerase chain reaction; SNP, single‐nucleotide polymorphism.

**FIGURE 1:**
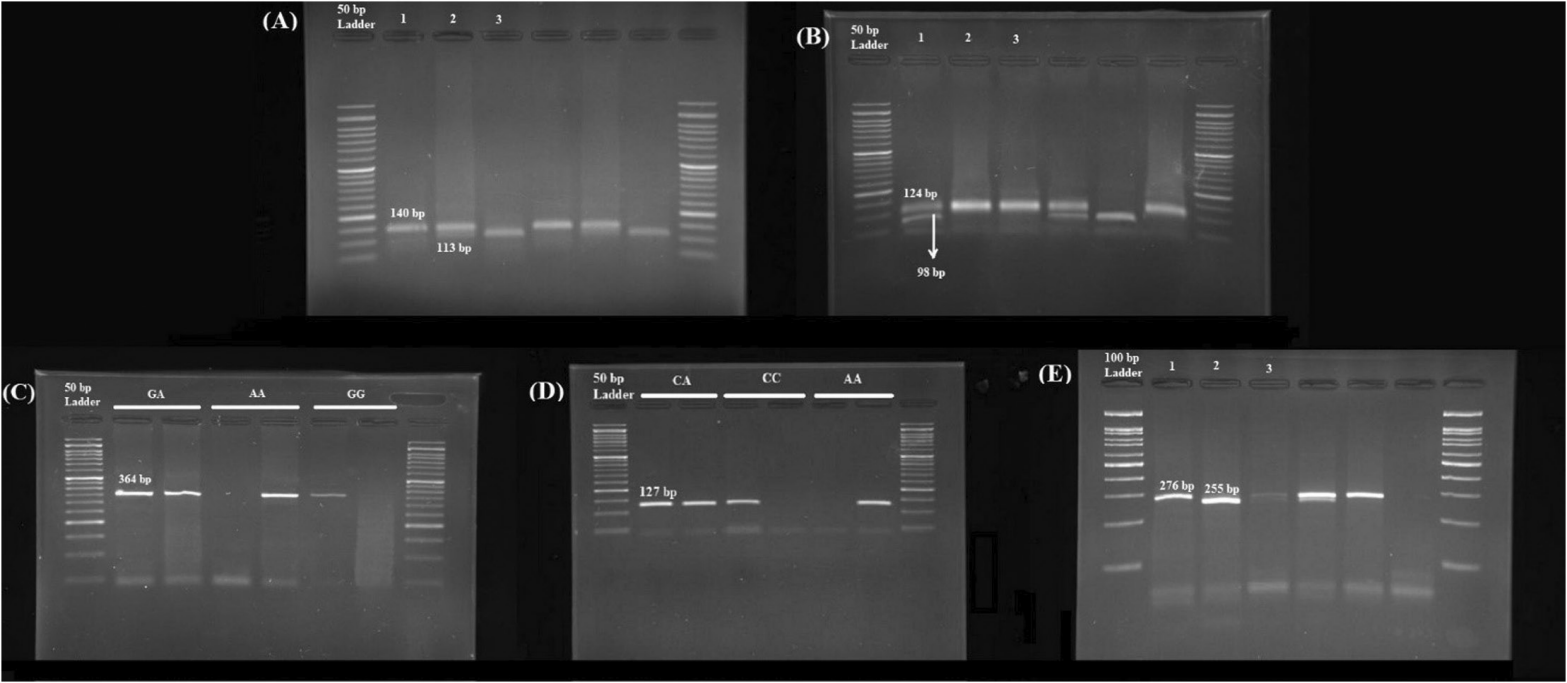
Gel electrophoretic separation of polymerase chain reaction products for genotyping *HOTAIR rs920778* (A), *rs4759314* (B), *rs12826786* (C), *rs1899663* (D), and *rs3816153* (E) polymorphisms.

Polymerase chain amplification was conducted in a total volume of 15 μL, containing 1 μL of genomic DNA (⁓10 ng/μL), 1 μL of each primer, 10 μL Taq 2x Master Mix (Ampliqon Inc., Denmark), and 2 μL sterile deionized water. PCR conditions included initial denaturation at 95°C for 5 min, followed by 35 cycles of denaturation at 95°C for 1 min, heating at annealing at temperatures (shown in Table 2 for each variant) for 40 s, extension at 72°C for 1 min, followed by a final extension at 72°C for 8 min. *MspI* and *AluI* restriction enzyme digestions were used for genotyping rs920778, rs4759314, and rs3816153, respectively. To visualize the PCR products, agarose gels were stained with a safe DNA stain (GreenViewer, Parstous Biotechnology, Iran). The remaining SNPs (rs12826786 and rs1899663) were genotyped using ARMS‐PCR. Figure 1 shows gel photographs of the examined polymorphisms were taken under ultraviolet (UV). There was approximately 100% agreement between the initial and replicate genotype results for almost 25% of the samples.

### 2.4 Statistical Analysis

The SPSS v. 22.0 software was utilized to analyze the data in this study. The chi‐square test assessed Hardy–Weinberg equilibrium (HWE) in control individuals. Moreover, Fisher's test and Student's *t*‐test were used to analyze categorical and continuous variables. This analysis also facilitated comparing the patients and control groups regarding allelic or genotypic frequencies. An odds ratio (OR) and a 95% confidence interval (CI) were calculated to assess the association between *HOTAIR* gene polymorphisms and CKD risk. Haplotypes and linkage disequilibrium (LD) were determined using the SHEsis online platform. Statistical significance was determined by a *p*‐value of less than 0.05.

## 3 Results

### 3.1 Clinical and Demographic Findings

Our findings showed that the mean age of the CKD patients (59 females and 91 males) was 51.13 ± 16.74 years, while the controls (71 females and 79 males) had a mean age of 49.64 ± 14.81 years (*p* > 0.05). Statistical analysis revealed no notable differences in age and gender distribution between patients and the healthy group (*p* > 0.05). However, as demonstrated in Table 1, a remarkable difference was noticed in terms of BMI between the studied groups (cases: 27.09 ± 4.08, controls: 24.44 ± 4.38; *p* < 0.001). Similarly, significant associations were found between serum creatinine and BUN levels in CKD cases and controls (*p*‐value <0.001). It is clear from the considerable difference between the two groups that the study groups were correctly selected based on serum creatinine levels.

### 3.2 Genetic Association Studies

The genotypic and allelic frequencies of *HOTAIR* SNPs in the CKD group and healthy individuals are summarized in Table 3. The allelic frequencies of the studied variations were consistent with HWE in the control group (*p*‐value for HWE >0.05 for all SNPs). Our finding revealed no statistical correlation between the frequency of the rs920778 variation and the risk of CKD neither in the genotypic nor the allelic inheritance models (*p* > 0.05 for all models). Regarding rs4759314, a diminished risk of CKD was observed under the codominant (AG vs. AA, OR = 0.19, 95% CI = 0.09–0.41, *p* < 0.001), dominant (AA + AG vs. GG, OR = 0.18, 95% CI = 0.09–0.39, *p* < 0.001), and overdominant (AG vs. AA + GG, OR = 0.20, 95% CI = 0.08–0.44, *p* < 0.001), and allelic (G vs. A, OR = 0.22, 95% CI = 0.12–0.40, *p* < 0.001) genetic patterns. Similarly, the T allele of rs3816153 conferred strong protection against CKD under the codominant (GT vs. GG, OR = 0.28, 95% CI = 0.15–0.55, *p* < 0.001), dominant (GG+GT vs. TT, OR = 0.55, 95% CI = 0.33–0.93, *p* = 0.025), and overdominant (GT vs. TT+GG, OR = 0.26, 95% CI = 0.14–0.51, *p* < 0.001) modes. On the other hand, rs12826786 exhibited an enhanced risk of CKD under the dominant (CC+CT vs. TT, OR = 1.66, 95% CI = 1.05–2.63, *p* = 0.031). Likewise, the T allele of this polymorphism increased the risk of CKD (T vs. C, OR = 1.50, 95% CI = 1.06–2.11, *p* = 0.021). Notably, the rs1899663 variant of the *HOTAIR* gene conferred an increased risk of CKD under codominant (TT vs. GG, OR = 2.24, 95% CI = 1.08–4.63, *p* = 0.030) as well as the allelic (T vs. G, OR = 1.46, 95% CI = 1.05–2.03, *p* = 0.023) modes of inheritance. We observed no statistically significant association between genotypes of the analyzed SNPs and CKD stages (Table S1).

**TABLE 3:** Genotypic and allelic distribution of *HOTAIR* polymorphisms in patients with CKD and healthy attendees.

Variant	Type	CKD (%)	Control (%)	Model	OR (95% CI)	*p*‐value*
rs920778 C/T	CC	49 (33.1)	39 (26.9)	Codominant1	0.64 (0.38–1.11)	0.111
	CT	62 (41.9)	78 (53.8)	Codominant2	1.06 (0.56–2.04)	0.850
	TT	37 (25.0)	28 (19.3)	Dominant	0.76 (0.46–1.25)	0.277
	HWE	0.057	0.323	Recessive	1.39 (0.79–2.42)	0.250
				Over‐dominant	0.62 (0.39–0.98)	0.084
	C	160 (54.1)	156 (53.8)	Allelic	1 [reference]	
	T	136 (45.9)	134 (46.2)	Allelic	0.99 (0.72–1.38)	0.991
rs4759314 A/G	AA	137 (92.6)	103 (70.1)	Codominant1	0.19 (0.09–0.41)	**<0.001**
	AG	9 (6.1)	36 (24.5)	Codominant2	0.21 (0.04–1.01)	0.052
	GG	2 (1.4)	8 (5.4)	Dominant	0.18 (0.09–0.39)	**<0.001**
	HWE	0.001	0.054	Recessive	0.25 (0.05–1.20)	0.082
				Over‐dominant	0.20 (0.08–0.44)	**<0.001**
	A	283 (95.6)	242 (82.3)	Allelic	1 [reference]	
	G	13 (4.4)	52 (17.7)	Allelic	0.22 (0.12–0.40)	**<0.001**
rs12826786 C/T	CC	61 (40.9)	79 (53.4)	Codominant1	1.57 (0.96–2.57)	0.075
	CT	64 (43.0)	53 (35.8)	Codominant2	1.94 (0.95–3.99)	0.070
	TT	24 (16.1)	16 (10.8)	Dominant	1.66 (1.05–2.63)	**0.031**
	HWE	0.302	0.127	Recessive	1.59 (0.81–3.14)	0.181
				Over‐dominant	1.35 (0.84–2.15)	0.210
	C	186 (62.4)	211 (71.3)	Allelic	1 [reference]	
	T	112 (37.6)	85 (28.7)	Allelic	1.50 (1.06–2.11)	**0.021**
rs1899663 G/T	GG	41 (27.3)	56 (37.6)	Codominant1	1.41 (0.84–2.35)	0.191
	GT	79 (52.7)	76 (51.0)	Codominant2	2.24 (1.08–4.63)	**0.030**
	TT	30 (20.0)	17 (11.4)	Dominant	1.60 (0.98–2.61)	0.060
	HWE	0.469	0.245	Recessive	1.88 (0.98–3.60)	0.056
				Over‐dominant	1.05 (0.67–1.66)	0.818
	G	161 (53.7)	188 (63.1)	Allelic	1 [reference]	
	T	139 (46.3)	110 (36.9)	Allelic	1.46 (1.05–2.03)	**0.023**
rs3816153 G/T	GG	116 (77.9)	99 (66.0)	Codominant1	0.28 (0.15–0.55)	**<0.001**
	GT	14 (9.4)	43 (28.7)	Codominant2	1.97 (0.82–4.71)	0.128
	TT	19 (12.7)	8 (5.3)	Dominant	0.55 (0.33–0.93)	**0.025**
	HWE	0.001	0.256	Recessive	2.52 (1.06–5.98)	**0.036**
				Over‐dominant	0.26 (0.14–0.51)	**<0.001**
	G	246 (82.5)	241 (80.3)	Allelic	1 [reference]	
	T	52 (17.5)	59 (19.7)	Allelic	0.86 (0.57–1.30)	0.468

*Note:* Bonferroni correction was applied. *p* < 0.05 was considered statistically significant (bold *p*‐values).

Abbreviations: CI, confidence interval; CKD, chronic renal insufficiency; OR, odds ratio.

* *p*‐value adjusted for BMI. Codominant1 and Codominant2 represent the heterozygous and homozygous codominant models, respectively.

### 3.3 Haplotype and Interaction Analyses

An interaction analysis of genotype combinations of studied SNPs with predisposition to CKD in Table 4 revealed no statistically significant associations (Table 4). We then conducted a haplotype analysis to examine the effects of all haplotypes of examined SNPs on CKD susceptibility (Table 5). Compared with the reference haplotype with the highest frequency among the controls (C_rs12826786_T_rs920778_G_rs1899663_A_rs4759314_G_rs3816153_), the CTGGG haplotype reduced the risk of CKD in our population by 84% (OR = 0.14, 95% CI = 0.03–0.66, *p* = 0.006). Based on our findings, *HOTAIR* SNPs were not in linkage disequilibrium (LD) (Figure S1).

**TABLE 4:** Interaction analysis of *HOTAIR* polymorphisms on CKD risk.

rs12826786 C/T	rs920778 C/T	rs1899663 G/T	rs4759314 A/G	rs3816153 G/T	CKD (%)	Control (%)	OR (95% CI)	*p*‐value*
CT	CT	GT	AA	GG	9 (8.4)	12 (12.6)	1 [reference]	
CC	CT	GT	AA	GG	12 (10.9)	11 (11.7)	1.45 (0.44–4.78)	0.541
CT	CT	GG	AA	GG	4 (3.6)	9 (9.6)	0.59 (0.14–2.55)	0.487
CT	CC	GT	AA	GG	6 (5.5)	4 (4.2)	2.00 (0.43–9.26)	0.380
CC	CC	GG	AA	GG	4 (3.6)	4 (4.2)	1.33 (0.26–6.83)	0.734
CC	CT	GT	AA	GT	1 (0.9)	4 (4.2)	0.33 (0.03–3.51)	0.354
CC	CT	GT	AG	GG	1 (0.9)	4 (4.2)	0.33 (0.03–3.51)	0.354
CC	CT	GG	AA	GG	7 (6.4)	3 (3.1)	3.11 (0.62–15.49)	0.164
CT	TT	GG	AA	GG	7 (6.4)	3 (3.1)	3.11 (0.62–15.49)	0.164
CT	CT	TT	AA	GG	6 (5.5)	3 (3.1)	2.67 (0.52–13.65)	0.240
CC	CC	GT	AA	GG	6 (5.5)	3 (3.1)	2.67 (0.52–13.65)	0.240
CC	TT	GT	AA	GT	3 (2.7)	3 (3.1)	1.33 (0.22–8.22)	0.760
CC	TT	GG	AA	GG	2 (1.8)	3 (3.1)	0.89 (0.12–6.48)	0.909
CC	CC	GT	AA	GT	1 (0.9)	3 (3.1)	0.44 (0.039–5.01)	0.513
CT	CT	GT	AA	GT	1 (0.9)	3 (3.1)	0.44 (0.039–5.01)	0.513
TT	CT	GT	AA	GG	6 (5.5)	2 (2.1)	4.00 (0.65–24.66)	0.128
CC	TT	GT	AA	GG	4 (3.6)	2 (2.1)	2.67 (0.40–17.91)	0.312
CC	CT	TT	AA	GG	2 (1.8)	2 (2.1)	1.33 (0.16–11.36)	0.796
TT	CC	GG	AA	GG	1 (0.9)	2 (2.1)	0.67 (0.05–8.55)	0.759
CC	CC	GG	AA	GT	1 (0.9)	2 (2.1)	0.67 (0.05–8.55)	0.759
CT	CC	GG	AA	GG	5 (4.5)	1 (1.0)	6.67 (0.66–67.46)	0.086
CT	CC	TT	AA	GG	5 (4.5)	1 (1.0)	6.67 (0.66–67.46)	0.086
CT	TT	GT	AA	GG	4 (3.6)	1 (1.0)	5.33 (0.50–56.24)	0.143
TT	CT	TT	AA	GG	2 (1.8)	1 (1.0)	2.67 (0.21–34.20)	0.448
CT	CC	GT	AG	GG	2 (1.8)	1 (1.0)	2.67 (0.21–34.20)	0.448

*Note:* Only the data in which the total of cases and controls was more than 2% have been included.

Abbreviations: CI, confidence interval; CKD, chronic renal insufficiency; OR, odds ratio.

* Bonferroni correction was applied and *p* < 0.01 was considered statistically significant.

**TABLE 5:** Haplotype analysis of *HOTAIR* polymorphisms on CKD risk.

rs12826786 C/T	rs920778 C/T	rs1899663 G/T	rs4759314 A/G	rs3816153 G/T	CKD (%)	Control (%)	OR (95% CI)	*p*‐value*
C	T	G	A	G	41 (14.0)	44 (15.8)	1 [reference]	
C	C	G	A	G	44 (15.1)	40 (14.3)	1.18 (0.64–2.16)	0.591
C	C	T	A	G	40 (13.7)	33 (11.9)	1.30 (0.69–2.43)	0.412
T	C	G	A	G	23 (7.9)	24 (8.7)	1.03 (0.50–2.10)	0.939
C	T	T	A	G	25 (8.6)	20 (7.2)	1.34 (0.65–2.77)	0.429
T	C	T	A	G	27 (9.2)	13 (4.7)	2.23 (1.01–4.89)	0.044
T	T	G	A	G	22 (7.6)	17 (6.1)	1.39 (0.65–2.98)	0.400
T	T	T	A	G	17 (5.9)	9 (3.2)	2.03 (0.81–5.05)	0.127
C	C	G	A	T	6 (2.0)	18 (6.5)	0.40 (0.14–1.13)	0.043
C	T	G	G	G	2 (0.7)	15 (5.4)	0.14 (0.03–0.66)	**0.006**
C	C	T	A	T	6 (2.0)	11 (4.0)	0.58 (0.20–1.73)	0.331
C	T	T	A	T	10 (3.5)	7 (2.5)	1.53 (0.53–4.40)	0.428
C	T	G	A	T	7 (2.4)	7 (2.5)	1.07 (0.35–3.32)	0.903
T	T	G	A	T	7 (2.4)	4 (1.4)	1.88 (0.51–6.89)	0.339
C	C	T	G	G	3 (1.0)	7 (2.5)	0.46 (0.11–1.90)	0.276
T	C	T	A	T	6 (2.0)	1 (0.4)	6.44 (0.74–55.80)	0.058
C	C	G	G	G	2 (0.7)	5 (1.8)	0.43 (0.08–2.34)	0.319
T	C	G	A	T	3 (1.0)	3 (1.1)	1.07 (0.20–5.62)	0.934

*Note:* Data were only included if the total of cases and controls exceeded 2%.

Abbreviations: CI, confidence interval; CKD, chronic renal insufficiency; OR, odds ratio.

* Bonferroni correction was applied and *p* < 0.01 was considered statistically significant (bold *p*‐value).

## 4 Discussion

CKD represents a pervasive global health condition with diverse etiologies. Key risk factors associated with the onset of kidney disease include diabetes, hypertension, cardiovascular disease, and a familial predisposition to kidney failure [26]. We found that rs4759314 confers a robust protective effect against CKD in heterozygotes with allelic, dominant, and codominant genotypes. Additionally, rs3816153 reduced the risk of CKD by 79% in individuals with the GT genotype compared to TT, 78% in those with TT compared to GG, 55% in individuals with GG+GT compared to TT, and 74% in individuals with GT compared to TT+GG. However, we found that T alleles in rs12826786 and rs3816153 and CC+CT genotypes were significantly associated with CKD. The haplotype C_rs12826786_T_rs920778_G_rs1899663_G_rs4759314_G_rs3816153_ was strongly associated with decreased CKD risk, although there was no linkage between the variants analyzed. The HOTAIR variants were not significantly associated with CKD stages.

Our study represents a novel investigation into the association between renal diseases and *HOTAIR* variations, highlighting a gap in the existing literature. Prior research has primarily concentrated on the impact of *HOTAIR* genetic variations on predisposition to diverse, complex diseases, some exhibiting comorbidity with CKD. In our prior investigation regarding the effect of lncRNAs in the context of diabetes, we found that *HOTAIR* rs920778, rs12826786, and rs4759314 SNPs had a significant correlation with T2DM. *HOTAIR* polymorphisms rs920778, rs12826786, and rs4759314 had significant correlations with T2DM risk in a population from southeast Iran, whereas rs1899663 showed a significant negative correlation. The rs4759314 gene was found to be significantly associated with both FBS and LDL‐c and rs920778 with HDL‐c in cases with T2DM. Based on the results of the haplotype analysis, the CCGG, CTTG, TGTA, and the TTTG genotype combination of rs920778/rs1899663/rs12826786/rs4759314 variations significantly increased T2DM risk by 1.47, 1.96, 2.81, and 4.80 folds, respectively, but that the four *HOTAIR* SNPs were not in LD [8]. In agreement with these findings, we did not observe LD between the studied variations, and rs12826786 was positively associated with CKD susceptibility in our study. However, the findings of Sargazi et al. showed an enhanced risk of T2DM under rs920778 and rs4759314 polymorphisms, which did not agree with our findings. The difference in research findings may be attributed to the heterogeneous etiology of diseases, specifically how T2DM might lead to CKD in its advanced stages [27]. Another factor influencing these discrepancies could be the discrepancy in sample sizes, with 500 T2DM cases enrolled compared to fewer CKD cases in the study population. Over the past few years, many studies, including genome‐wide association studies (GWAS), have offered a hypothesis‐free method for detecting prevalent genetic variations that may contribute to the genetic predisposition of prevalent diseases like CKD and T2DM [28, 29, 30]. In another genetic association study, Mohammadpour et al. reported that *HOTAIR* polymorphisms are associated with preeclampsia, a pregnancy disorder that is commonly characterized by hypertension and proteinuria after 20 weeks [31]. This is important since several reports have established a sizable association between preeclampsia and End‐Stage Renal Disease (ESRD) [32, 33, 34]. A recent report by Kim et al. illustrated that *HOTAIR* rs4759314, rs1899663, and rs12826786 genotype combinations strongly affect coronary artery disease occurrence [14]. Yet, no study has investigated the correlation between *HOTAIR* gene variants and CKD incidence. Our study examined a cohort of Iranian patients suffering from CKD in conjunction with a control group to determine which *HOTAIR* gene polymorphisms are associated with CKD risk. In our population, the risk of CKD development was significantly associated with *HOTAIR* polymorphisms rs4759314, rs12826786, rs1899663, and rs3816153.

Numerous potential mechanisms contributing to the risks of CKD can be elucidated as follows: First, metabolic diseases and obesity, prevalent factors in hypertensive, and diabetic patients have been implicated in the progression of nephropathy [35]. Second, an observed increase in peripheral arterial resistance and resistant hypertension has been linked to an elevated risk of end‐stage renal diseases [36]. Additionally, it was reported that the upregulation of the renin–angiotensin–aldosterone system and sodium transporters had the potential to disrupt the glomerular structure, ultimately resulting in albuminuria and diminished nephron function [37]. The significant role of reactive oxygen metabolites in the progression of diabetic kidney disease, facilitated by the endoplasmic reticulum stress, has been highlighted in earlier studies [38].

Various pathogenic processes, including the progression of chronic kidney disease, are linked to the dysregulation of lncRNAs [39]. LncRNAs are RNA molecules lacking coding potential and having a length greater than 200 nucleotides. In addition to modulating protein functions and epigenetic modification, these molecules act as enhancer RNAs and modulate small RNA functions. Notably, lncRNAs display a highly tissue‐specific expression pattern, indicative of their intricate involvement in cellular processes [11]. Additionally, lncRNAs are regulated in adult stem/progenitor cells, especially in the human kidney. For instance, the lncRNA HOTAIR has been identified as a factor controlling self‐renewal and cell senescence [40]. HOTAIR supports these cells in secreting elevated amounts of α‐Klotho, an anti‐aging protein that exerts influence on the surrounding tissues, thereby modulating the aging process in the kidneys. Subsequently, Klotho influences diverse signaling pathways, such as p53/p21, Wnt, Insulin, cAMP, protein kinase C, and TGF‐β [41]. Interestingly, autosomal dominant polycystic kidney disease (ADPKD) patients with cystic kidneys had lower levels of HOXB3‐AS1. According to the study, RNA‐seq analysis on cystic kidneys of ADPKD mutant mice detected dysregulated lncRNAs and confirmed that Hoxb3os lncRNA alters mTOR signaling and mitochondrial respiration and functions as a negative regulator [42]. The correlation between polymorphisms in mTOR pathway genes and kidney disorders has been extensively documented in numerous studies [43].

In recent developments, a microarray analysis conducted on monocytes from patients with IgA nephropathy [44] and a systems biology study [45] have collectively identified over 200 differentially expressed lncRNAs in peripheral blood mononuclear cells, implicating their potential involvement in the pathophysiology of IgA nephropathy. Notably, the research posited that HOTAIR emerged as the principal lncRNA regulating differentially expressed genes and microRNAs in the context of IgA nephropathy. Furthermore, genetic variants within genes associated with the innate immune system have been demonstrated to be linked with the progression of CKD in patients with IgA nephropathy [46].

The protective role of HOXA cluster antisense RNA 2 (HOXA‐AS2) in septic‐induced acute kidney injury (SI‐AKI) was observed by M'Baya‐Moutoula et al. multi‐omics study [47]. This protective effect was attributed to the modulation of miR‐106b‐5p and the inhibition of the Wnt/β‐catenin and NF‐κB pathways. Additionally, Shen et al. reported that HOTAIR, by inhibiting miR‐22, facilitated cell death. Suppression of HOTAIR, conversely, resulted in improved renal function [48]. Mutations in the genes encoding critical mediators of the Wnt/β‐catenin pathway have been associated with susceptibility to CKD [49].

Subsequently, a study by Jiang et al. demonstrated that overexpression of HOTAIR led to a reduction in renal damage, inflammation, and cell death within the kidney, achieved through downregulation of the miR‐34a/Bcl‐2 signaling pathway [50]. They observed a decline in the expression of HOTAIR, which correlated with decreased serum serine and blood urea nitrogen levels and diminished signs of apoptosis in kidney tissue. A simultaneous increase in Bcl‐2 protein levels and a reduction in miR‐34a were connected to these effects. The effects of miR‐34a on apoptotic processes can be explained by the direct target of Bcl‐2, which has been recognized as an antiapoptotic factor. There are many studies confirming the impact of polymorphisms in genes associated with the apoptosis pathway and inflammation on the development of CKD [51].

The application of SNPs in personalized medicine in kidney diseases has great potential since it will help optimize patient‐targeted treatment and concentrate healthcare resources [52]. SNPs are responsible for interindividual variation in the risk of disease, the response to treatments, and the metabolism of medicines [53]. Some SNPs have been linked to an increased risk of developing CKD or kidney stones [54]; therefore, by evaluating the SNP profile of such a person, healthcare providers can identify those at a higher risk and prescribe measures to prevent the development of CKD. It is also possible that these variations may directly affect the reaction to specific drugs that are used for kidney transplant recipients or antihypertensive medications for kidney disease patients [55]. To achieve this, healthcare providers can use SNPs linked to drug metabolism or drug targets to formulate specific drugs that will yield maximum benefit to patients with minimal side effects. This is where the SNP profile of an individual can help clinicians in choosing the right treatment option and also in giving the proper dosages of medication to achieve the best possible results and determining the appropriate dose of a medication to be administered to an individual concerning their metabolism rate to prevent drug toxicity or failed treatment [56].

While genetic factors, particularly polymorphisms within lncRNA genes, have been identified as contributors to an elevated risk of kidney disease [57], our study represents the initial report elucidating the association between *HOTAIR* genetic variants and the risk of CKD. Our study is limited in certain ways, which needs to be acknowledged. There may not be enough diversity in the Iranian population because of the relatively small sample size and the fact that it was collected exclusively from one location. We encountered difficulty determining the exact genotype in a small subset of samples. Despite reanalysis, a few genotypes could not be determined due to technical issues and limited DNA sample volume. Yet, we are confident that this slight difference did not affect the analysis process, as there are still enough samples for the case and control groups to ensure sufficient statistical power for the analyses. More studies are required to validate the findings of this study and to use a higher number of participants of various ethnicities and more accurate genotyping techniques. We have demonstrated that *HOTAIR* gene polymorphisms are significantly correlated with CKD risk among a sample of Iranian women.

## 5 Conclusion

This study, the first to investigate variations in the *HOTAIR* gene among Iranian individuals, suggests that this gene may influence CKD development. Identifying these genetic markers could improve both the diagnosis and treatment of this condition. These gene variants may prove helpful as molecular biomarkers for a broader range of clinical purposes if studies involve a larger sample size and diverse ethnic groups. Future research should also consider investigating additional genetic variants and exploring the functional consequences of the identified associations to further understand the mechanisms underlying CKD and enhance the potential for targeted therapeutic interventions.

## Ethics Statement

The local Ethics committee of Zahedan University of Medical Sciences approved the protocol of the current study (Ethical code: IR.ZAUMS.REC.1400.376). The webpage of the ethical approval code is available at https://ethics.research.ac.ir/ProposalCertificateEn.php?id=243644&Print=true&NoPrintHeader=true&NoPrintFooter=true&NoPrintPageBorder=true&LetterPrint=true. Informed consent was taken from all enrolled subjects.

## Conflicts of Interest

The authors declare no conflicts of interest.

## Supporting information


**TABLE S1.** Association between *HOTAIR* SNPs with staging CKD patients.
**FIGURE S1.** Pairwise LD analysis of HOTAIR rs12826786, rs920778, rs1899663, rs4759314 and rs3816153 polymorphisms. No LD was observed between the examined variations.

## Data Availability

The data that support the findings of this study are available from the corresponding author upon reasonable request.
