# Supplementary material for: A Study on Associations of Long Noncoding RNA HOTAIR Polymorphisms With Genetic Susceptibility to Chronic Kidney Disease
Source: J Clin Lab Anal. 2024 Jul 3;38(11-12):e25086. doi: 10.1002/jcla.25086 (PMC11252834; doi:10.1002/jcla.25086)
Supplement: Supplementary file 1 — TABLE S1. Association between HOTAIR SNPs with staging CKD patients. FIGURE S1. Pairwise LD analysis of HOTAIR rs12826786, rs920778, rs1899663, rs4759314 and rs3816153 polymorphisms. No LD was observed between the examined variations. [file JCLA-38-e25086-s001.docx]

**Table S1.** Association between *HOTAIR* SNPs with staging CKD patients.

| **Variant** | **Genotype** | ***stage I*** | ***stage II*** | ***stage III*** | ***stage IV*** | ***stage V*** | ***p-value*** |
| --- | --- | --- | --- | --- | --- | --- | --- |
| rs920778 C/T | CC  CT+TT | 17  27 | 15  18 | 12  36 | 2  13 | 3  5 | 0.065 |
| rs4759314 A/G | AA  AG+GG | 42  2 | 30  3 | 44  4 | 13  2 | 8  0 | 0.541 |
| rs12826786 C/T | CC  CT+TT | 14  30 | 12  21 | 24  25 | 9  6 | 2  6 | 0.094 |
| rs1899663 G/T | GG  GT+TT | 11  34 | 6  27 | 16  33 | 7  8 | 1  7 | 0.239 |
| rs3816153 G/T | GG  GT+TT | 34  10 | 27  6 | 38  11 | 11  4 | 6  2 | 0.758 |


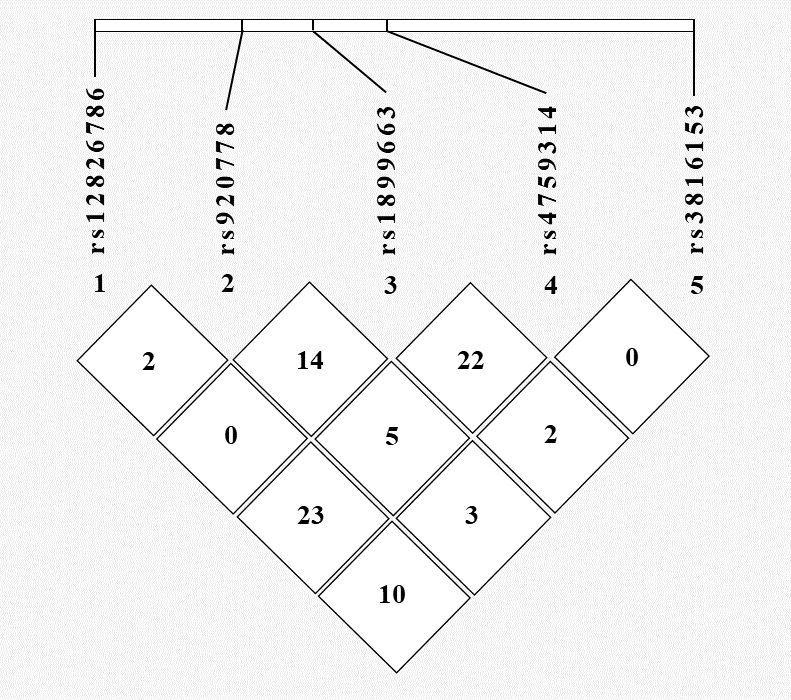


**Figure S1.** Pairwise LD analysis of *HOTAIR rs12826786*, *rs920778*, *rs1899663*, *rs4759314* and *rs3816153* polymorphisms**.** No LD was observed between the examined variations.
